# Exploring quantum Griffiths phase in Ni_1−*x*_V_*x*_ nanoalloys

**DOI:** 10.1038/s41598-017-01423-x

**Published:** 2017-04-21

**Authors:** Priyadarsini Swain, Suneel K. Srivastava, Sanjeev K. Srivastava

**Affiliations:** 1 Department of Physics, Indian Institute of Technology Kharagpur, Kharagpur, 721302 India; 2 Department of Chemsitry, Indian Institute of Technology Kharagpur, Kharagpur, 721302 India

## Abstract

Metallic Ni_1−*x*_V_*x*_ alloys exhibit a ferromagnetic to paramagnetic disordered quantum phase transition in bulk. Such a phase transition is accompanied by a quantum Griffiths phase (QGP), featuring fractional power-law temperature dependences of physical variables, like magnetic susceptibility and specific heat, at low temperatures. As nanoparticles (NP’s) usually exhibit properties significantly different from their bulk counterparts, it is intriguing to explore the occurrence of quantum Griffiths phase in Ni_1−*x*_V_*x*_ nanoalloys. NP’s of Ni_1−*x*_V_*x*_ (0 ≤ *x* ≤ 0.17) alloys are prepared by a chemical reflux method. The structure and composition of the nanoalloys are determined by X-ray diffraction, X-ray photoelectron spectroscopy and electron microscopy techniques. Metallicity of the samples has been ensured by electrical resistivity measurements. DC magnetization results suggest that ferromagnetism persists in the NP’s until *x* = 0.17. Low-temperature upturns in magnetic susceptibility and heat capacity hint at critical fluctuations evolving with V-doping. The fluctuations might stem from isolated Ni-clusters within the ferromagnetic NP, indicating a QGP region ranging from *x* = 0.085 to *x* ≫ 0.17.

## Introduction

Phase transitions are ubiquitous and occur in a wide variety of materials. Conventionally, phase transitions are driven by thermal fluctuations that destroy the order as the sample is warmed through its transition. Phase transitions are further divided into two classes: (i) first-order phase transitions, where the first derivative of free energy is discontinuous at the transition temperature *T*_*c*_; this features phase coexistence and involves latent heat at *T*_*c*_^1^, and (ii) second-order or continuous phase transitions, where the discontinuity is in the second derivative of free energy, like in the gradient of magnetization in the case of ferromagnetic (FM) to paramagnetic (PM) phase transition^1^. The critical point T_*c*_ in such a transition exhibits strong fluctuations in the system at large length scales characterized by a correlation length *ξ* which diverges as *ξ* ~ |(*T* − *T*_*c*_)|^−*ν*^ ^2^ (*T*: temperature, *ν*: correlation length critical exponent). Further, these fluctuations close to criticality are also slow and are characterized by a correlation time *ξ*_*t*_ which also diverges at *T*_*c*_^2^. These power-law singularities in the length and time scales at a critical point lead to power-law singularities also in observable quantities^2^. At an FM critical point, specific heat *c*, the order parameter, (viz., the magnetization *M* defined for *T* < *T*_*c*_), and magnetic susceptibility *χ* (defined for *T* > *T*_*c*_) follow the relations *c* ~ |(*T* − *T*_*c*_)|^−*α*^, *M* ~ |(*T* − *T*_*c*_)|^*β*^ and *χ* ~ |(*T* − *T*_*c*_)|^−*γ*^ with critical exponents *α*, *β* and *γ*, respectively^1, 2^. Since the fluctuations at all length scales become equally important at the criticality because of the divergence of *ξ*, many of the microscopic details of the system become irrelevant; only the order parameter and the dimensionality of the system are relevant in determining the critical exponents. This gives rise to the principle of *universality*, according to which every phase transition belongs to a universality class of transitions with the same critical exponents, even though the microscopic details of the systems can be completely different^1, 3^. The critical exponents for different universality classes, like Ising, Heisenberg, etc., can be found easily in the literature^1, 3^.

Thermal-fluctuation driven phase transitions as described above are known as classical phase transitions. In certain situations, the critical temperature can be suppressed by varying a non-thermal control parameter *g*, like pressure, magnetic field or chemical composition. The system then undergoes a zero-temperature second-order phase transition, a critical value *g*_*c*_ of *g* separating an ordered quantum state from a disordered one. Here, quantum fluctuations determined by Heisenberg’s uncertainty principle destroy the order as *g* is varied through *g*_*c*_, and hence such a phase transition is called a quantum phase transition (QPT)^1, 3, 4^. The system then becomes critical with respect to *g* in the vicinity of *g*_*c*_, also known as quantum critical point (QCP)^2^. As a consequence, the correlation length attains a singularity *ξ* ~ |(*g* − *g*_*c*_)/*g*_*c*_|^−*ν*^ ^2^ and the system exhibits unconventional physical behaviour, like a non-Fermi liquid (NFL) phase, characterized by non-universal power-law temperature dependences of physical observables, around QCP^4^. The QCP controls a large portion of the *T* − *g* phase diagram even at non-zero temperatures. Among the many systems shown to exhibit QPT, a number of metallic ferromagnets constitute one class. A recent article by Brando *et al*.^5^ presents an extensive review on this subject. Out of these metallic ferromagnets, binary alloys based on the itinerant electron ferromagnet Ni are of particular interest because the *T*_*c*_ of Ni can be suppressed to absolute zero on alloying it with a critical concentration *x*_*c*_ of a non-magnetic *d*-element, like Pd^6^, Pt^7^, Rh^8^, or V^9^, and the binary nature of the alloy is structurally simple. Two extreme cases based on the amount of compositional disorder can be identified in these systems: (i) Ni_*x*_Pd_1−*x*_, with weak disorder at the QCP (*x*_*c*_ ~ 0.026); a study of this QPT by Nicklas *et al*. revealed (*x* − *x*_*c*_)^3/4^ dependence of *T*_*c*_ on *x* above *x*_*c*_, an unconventional *T* ln *T* dependence of low-*T* specific heat, and a power-law temperature dependence of low-*T* resistivity with a NFL exponent of temperature^6^, and (ii) Ni_1−*x*_V_*x*_, with a substantially large quenched disorder at the QCP (*x*_*c*_ ~ 0.114)^9^. In this, the non-magnetic impurity (V) removes the magnetic spins of Ni from the lattice randomly. In addition, a V impurity reduces the moments on the neighbouring Ni atoms strongly (by about 90%)^5^. In case of QPT’s, this situation is described by the so-called site-diluted transverse-field Ising model, as described by Vojta^2^. At the percolation threshold which happens to be roughly the QCP for this alloy system, the lattice comprises of infinite PM clusters accompanied with only finite-sized FM clusters, resulting in the destruction of the long-range FM order. However, smaller magnetic Ni cluster inclusions known as rare-regions are present in the infinite PM clusters due to statistical fluctuations in the distribution of V impurities in the lattice^2^. Because the rare-regions are of finite size, they can not undergo a true phase transition by themselves, they just act as large superspins. The parameter region in the *T* − *x* phase diagram where such rare-regions exist but long range order has not yet developed is called the Griffiths phase or the Griffiths region^2^. The singularities in thermodynamic quantities at *x*_*c*_ then extend also in the Griffiths region and lead to low-*T* dependences of observables as *c* ~ *T* ^*λ*^ and *χ* ~ *T* ^*λ*−1^, where *λ* is the common parametrized exponent^2, 9, 10^. The non-universal Griffiths exponent *λ* changes continuously throughout the Griffiths phase: it takes the value 0 at the QCP (*x*_*c*_) and increases with distance from the QCP towards the PM side^2, 9^. Thus, *λ* is a proper fraction (0 < *λ* < 1) in a QGP region. Ubaid-Kassis *et al*.^9^ in their work on QGP in bulk Ni_1−*x*_V_*x*_ alloys have plotted rather (1w-*T* dependences of observables*λ*) as a function of *x* and have shown that it decays exponentially from the value 1 at QCP to 0 towards pure PM phase. Further, if the rare regions themselves become infinitely large, or are coupled to an infinite bath leading to quantum dissipation, the QPT would get smeared^2^.

So far, most of the existing studies on QPT’s have been performed on bulk materials. Downscaled materials like nanoparticles, on the other hand, have been excluded from such studies probably because they possess inherent large disorders in the form of large surface areas^11^, and hence quantum fluctuations are likely to be smeared or washed out altogether. The magnetic behaviour of magnetic NP’s, wherein a FM-PM QPT could be possible, is further complicated^11^, and NFL behaviour or Griffiths singulartities would be difficult to extract. Nevertheless, it would be desirable to explore the occurrence of QPT and related phases in NP’s. It is anticipated that possible existence of a QCP could be integrated with the quantum physical phenomena exhibited by nanoparticles^11, 12^ and exploited technologically. This inquisitiveness led the authors earlier to investigate the occurrence of QPT in Ni_*x*_Pd_1−*x*_ nanoalloy system^13^, which was found to exhibit a QPT in spite of not showing any NFL behaviour. If one rather wants to explore QGP’s, wherein structure and magnetism are correlated as described above, the intricacies of magnetic NP structure and related magnetism vis-a-vis QGP scenario have to be examined first. There are essentially three features possessed by NP’s: (i) particle dimensions *r* on the same length scales as the characteristic lengths (e.g., domain wall size in ferromagnets), (ii) broken translational symmetry, i.e., disorder at the NP surface, and (iii) a high ratio of surface atoms to bulk atoms^14, 15^. For *r* less than a critical size determined by material properties, the NP is a single-domain particle, and the Stoner-Wohlfarth model can be used to understand the magnetism of a collection of such NP’s^15^. This model assumes that the particles have an uniaxial anisotropy, are of perfect spherical shape, and the individual spins in an NP rotate in unison like a large superspin. An exchange interaction *J* operates between spins in a single-domain NP to keep all the NP moments in one direction to make the NP equivalent to a superspin. In absence of an external magnetic field, a superspin can have its orientation either parallel or antiparallel to the direction of the anisotropy axis. The superspin can flip between these two states with a frequency $\tau ={\tau }_{0}{{\rm{e}}}^{KV/{k}_{B}T}$, where *K* is the magnetocrystalline anisotropy, *V*, the particle volume, *k*_*B*_, the Boltzmann constant, and *τ*_0_ is the elementary spin-flip time (~10^−12^−10^−9^ s)^14, 15^. At high-*T*, the superspins flip much faster than the experimental time scale, while at low-*T* they are much slower. Because of the extremely small flip rate at low temperatures, the superspins are ‘frozen’ or ‘blocked’. If the experimental time scale is *τ*_*m*_, then the blocking of the superspins starts at a temperature *T*_*B*_ = *KV*/{*k*_*B*_ ln(*τ*_*m*_/*τ*_0_)} ≈ *KV*/(30 *k*_*B*_), known as blocking temperature^14–16^, on cooling. Above *T*_*B*_, therefore, an ensemble of the NP superspins is in a PM state called superparamagnetic (SPM) state. If the ensemble is cooled in a small external field (field cooled or FC), the FC magnetization of the system will increase like a normal paramagnet down to *T*_*B*_, and then will saturate gradually at lower temperatures like a normal ferromagnet. If, on the other hand, the ensemble is cooled in absence of field (zero-field cooled or ZFC), the individual superspins will remain blocked randomly either parallel or antiparallel to the anisotropy direction at *T* = 0 (or at the lowest temperature accessible in the experiment). If the magnetization is measured now in a small external field, it would be zero at 0 K or nearly zero at the lowest experimental temperature. If the temperature is now raised while the field is still applied, the ‘antiparallel’ superspins get progressively forced by the field to become ‘parallel’, and the magnetization increases till *T*_*B*_ is reached; beyond that, it follows the FC magnetization curve. This leads to the splitting of FC and ZFC magnetization ideally at *T*_*B*_^14, 15^. Therefore, it is anticipated that the NP ensemble is in a blocked ferromagnetic (BFM) state, denoting blocked state in ZFC and FM behaviour in FC magnetization, below *T*_*B*_. For an NP ensemble with a distribution of particle size or with interparticle exchange interactions, there appears a peak in the ZFC magnetization at *T*_*B*_, and the FC-ZFC splitting takes place at a slightly higher temperature^14, 15^. Further, beyond the FM-PM transition temperature, the spins even in an NP fluctuate randomly and the system is in a true PM state. Thus, the magnetism of NP’s is more complex than the corresponding bulk which may feature just two (FM and PM) states, making it difficult to extract QGP in NP’s, if present. The second feature, viz., the broken translational symmetry at the NP surface results in a disordered surface layer comprising of vacancies, broken bonds and lattice strain. This structural disorder also produces a surface spin disorder characterized by a magnetocrystalline anisotropy *K*_*s*_ different from the anisotropy *K* in the core beneath the disordered surface^14^. Within the Stoner-Wohlfarth model, this leads to various canted spin structures at the NP surface^14^. Mössbauer measurements have shown that the hyperfine field at magnetic NP surface are less than that in the core^14^. Further, from a report on relation between hyperfine field and magnetic exchange interaction in metallic AuFe alloys, it can be deduced that a reduction in the hyperfine field is associated with a reduction in the exchange interaction^17^. Thus, for a metallic FM nanoparticle, one may expect the exchange interaction at the surface *J*_*s*_ to be less than the *J*-value in the core. The NP surface would then become like another FM component of the NP with a lower *T*_*c*_ than the core. This would add further complexity to the magnetic structure of a NP ensemble.

It is thus quite intriguing to explore whether an FM binary alloy system like Ni_1−*x*_V_*x*_ which possesses a QGP in bulk does so also when in the form of nanoparticles. As a matter of fact, Ni-V alloy nanoparticles have hitherto not been synthesized and studied for magnetic properties, or even for any application, to the best of authors’ knowledge. So, any kind of investigation, including the exploration of a QGP, on this nanoalloy system requires, as a pre-step, finding a method to prepare these nanoalloys. A versatile approach of preparing nanoparticles of materials is the existing multitude of bottom-up chemical methods. We are actively engaged in nanoparticle syntheses via such routes, a recent work being on microwave trapping by hollow polypyrrole particles^18^. Ni nanoparticles^19–22^ and Ni-V alloy microparticles^23^ have earlier been synthesized and shown to possess FM^19, 20^, SPM^21^, photocatalytic^22^ and hydrogen-storage^23^ properties. In a recent study^13^, we prepared rather Ni_*x*_Pd_1−*x*_ NP’s by a chemical reflux method. A chemical reflux method can also be tried for preparing Ni_1−*x*_V_*x*_ NP’s. Since structural properties of NP’s, as discussed above, are intricately connected with their magnetic properties, especially in connection with the existence of a QGP, it would be crucial as the next step to do a rigorous structural characterization of the prepared NP’s before studying the magnetic properties and resulting possible QGP.

In this work, we aim at exploring the presence of QGP in Ni_1−*x*_V_*x*_ nanoalloy system. For preparation of the nanoalloys, the chemical reflux method used to synthesize Ni_*x*_Pd_1−*x*_ nanoalloys in our previous work^13^, but with an appropriately modified set of chemicals, was adopted. Nanoparticles of Ni, V and Ni_1−*x*_V_*x*_, with *x* in the vicinity of *x*_*c*_, were prepared for the investigations. After determining the sizes, phases and compositions by different microscopic and spectroscopic techniques, the existence of QGP was explored primarily by dc magnetization and heat capacity measurements.

## Results and Discussion

**Figure 1:**
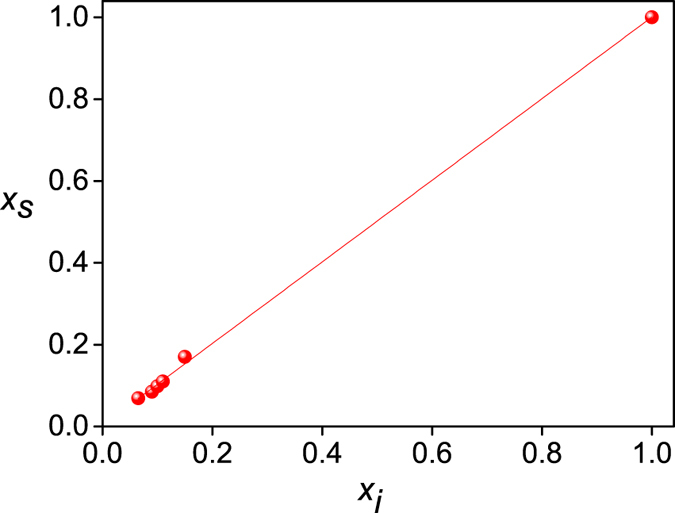
Variation of the composition *x*_*s*_ determined from EDAX with the initial composition *x*_*i*_.

**Figure 2:**
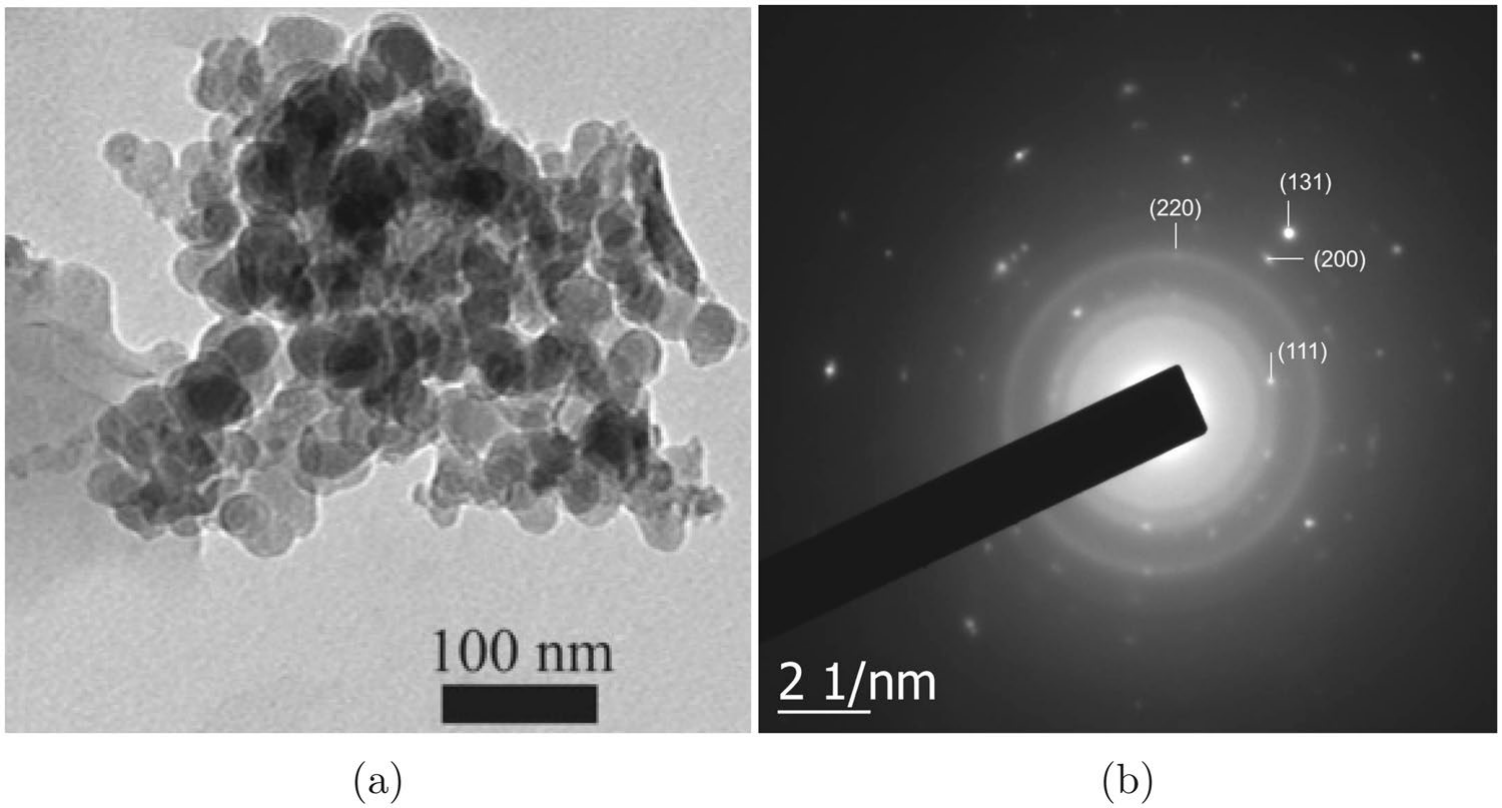
(**a**) HRTEM image of pure Ni NP’s, and (**b**) the corresponding SAED patterns. Initial four rings are indexed with a tag on a bright spot of a ring.

Motivated by our earlier work on Ni_*x*_Pd_1−*x*_ NP’s, we prepared Ni_1−*x*_V_*x*_ (*x* = 0, 0.069, 0.085, 0.098, 0.11, 0.17 and 1) nanoparticles by a chemical reflux method as detailed in the ‘Methods’ section later. A surfactant (diethanolamine) has been used in preparing the NP’s. The surfactant is likely to get coated on each NP’s and isolate them from each other. The coating also is likely to help prevent inter-particle magnetic interactions so that the NP’s do not agglomerate to become very big particles. As the first step of structural characterization, energy dispersive analysis of X-rays (EDAX) is performed on the samples. The variation of EDAX determined composition *x*_*s*_ with the initial composition *x*_*i*_ is plotted in Fig. 1. As is evident from the figure, *x*_*s*_ shows a linear variation (*x*_*s*_ = 0.0034 + 0.997 *x*_*i*_) with *x*_*i*_, and hence confirms that the stoichiometries taken during the syntheses are essentially the same as in the finally synthesized samples. Henceforth, *x*_*s*_ will be taken for *x* in Ni_1−*x*_V_*x*_. Field-emission scanning electron microscopy (FESEM) has been performed next on the samples. FESEM images, along with the corresponding size distributions, of pure Ni and Ni_1−*x*_V_*x*_ alloy samples are provided in Fig. S1 of the Supplementary information. The average particle sizes according to the FESEM images range between 18 to 33 nm for the alloys of different compositions. Considering the critical size ~21 nm for Ni as reported in literature^24^, the present NP’s can be assumed to be in the range of single-domain particles. To analyze the particles further, high-resolution transmission electron microscopy (HRTEM) has been performed on three representative samples with *x* = 0 (pure Ni), 0.098 and 0.11, along with their selected area electron diffraction (SAED) patterns. HRTEM image and SAED pattern for pure Ni nanoparticle are shown in Fig. 2, while those for *x* = 0.098 and 0.11 are provided in Fig. S2 of the Supplementary information. Atomic resolution images could not be taken for the NP’s because of their magnetic nature, as will also be evident from magnetization results being discussed later. The apparent NP agglomeration while still maintaining the isolation, as can be seen from the images (Fig. 2(a), S2(a), S2(c)), also suggests that the NP’s are coated with the surfactant and are of magnetic nature^25^. In the absence of atomic resolution images, however, the coatings can not be observed in the HRTEM images. The SAED pattern in Fig. 2(b) comprises of concentric rings (indicative of randomly oriented NP crystallites) and some dots (characteristic of single-crystal nature of NP’s) lying on these rings. It can thus be conjectured that the NP’s are essentially crystalline in nature, but various NP’s are oriented randomly. Initial four rings of the SAED pattern are indexed in Fig. 2(a) with the (hkl) flag on a diffraction spot of the ring. The (hkl) values correspond to fcc lattice, as expected. Other SAED patterns (Fig. S2(b) and S2(d), not indexed) also show the same crystal structure of the alloy NP’s.

**Figure 3:**
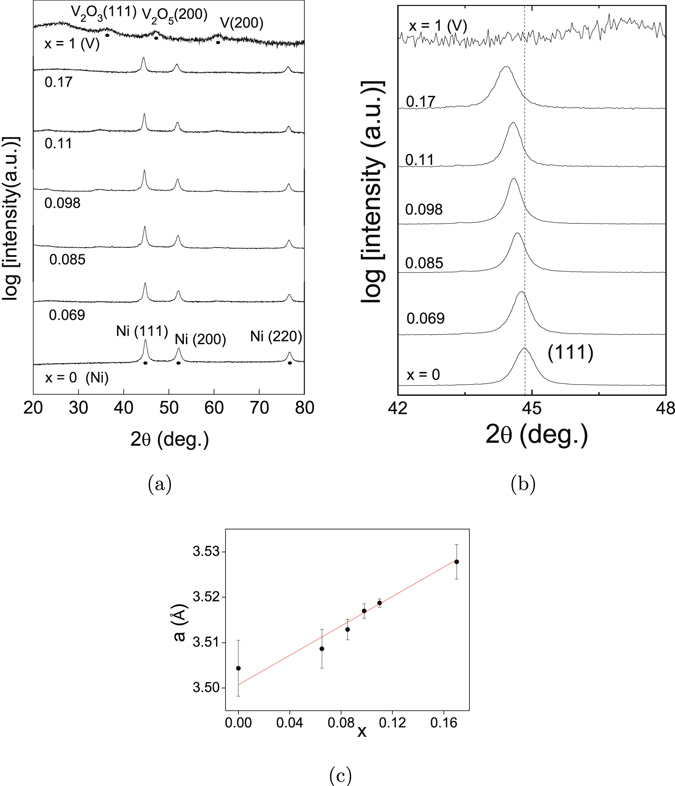
(**a**) XRD patterns of Ni_1−*x*_V_*x*_ samples. Details are explained in the main text. (**b**) Magnified view of the XRD patterns around (111) peak. The dotted vertical line is for reference. (**c**) Variation of lattice constant *a* with composition *x*.

As a supplement to the EDAX results on the crystallinity and crystal structures of the NP’s, X-ray diffraction (XRD) has also been performed on them. Figure 3(a) shows the XRD patterns of all the Ni_1−*x*_V_*x*_ (0 ≤ x ≤ 1) samples studied. The pattern for pure Ni NP’s consists of three clean peaks at 44.50°, 51.84° and 76.36°. According to the Joint Committee on Powder Diffraction Standards (JCPDS) data, these peaks correspond to (111), (200) and (222) reflections of fcc Ni, but relatively displaced towards 2*θ* values higher than the corresponding bulk. This observation reveals that the Ni NP’s are pure in phase and are of a lattice constant (3.499 Å) smaller than the corresponding bulk (3.524 Å) value. Reduction in the lattice constant in metals on decreasing the size happens because the enhanced surface energy due to an increased surface area tends to contract the lattice^26^. The change Δ*a* in the lattice constant *a* follows an inverse relation Δ*a* = −*a*/(1 + *Kr*), where *K* is a constant determined by the rigidity modulus and surface energy per unit area of the bulk^26^. The addition of V atoms to Ni up to *x* = 0.17 does not alter the three-peak structure, except that these peaks progressively shift towards lower 2*θ* values. A magnified view of the data around the (111) peak is displayed in Fig. 3(b) to demonstrate the shifts. Further, essentially no additional peaks appear on V incorporation in Ni. This suggests that the alloy NP’s also are in fcc phase and hence are in the form of Ni-V solid solutions. The alloy lattice constants *a*(*x*), as determined from the (111) peak positions, are plotted as a function of *x* in Fig. 3(c), and seem to vary linearly with *x*. A linear fit yields *a*(*x*) = 3.4964 + 0.1898 *x*. Assuming a close-packed accommodation of the impurities, and hence that *a*(*x*) is proportional to the weighted average of the atomic radii *r*_*V*_ and *r*_*N*_ *i* of V and Ni, respectively, the ratio *r*_*V*_/*r*_*N*_ *i* from the fitting parameters comes out to be 1.054. This value is close to 1.033, the covalent radius ratio for V and Ni. This once again confirms our earlier inference that the alloy NP’s are in the structural form of Ni-V solid solutions. The linear increase of the lattice constant up to *x* = 0.17 suggests that V has a solid solubility of at least 17% in Ni when in the form of NP’s. This is more than the bulk solid solubility limit (≤14%) according to the Ni-V phase diagram^27^. Solid solubilities in nanodimensions have earlier also been reported to be enhanced with respect to bulk^28^, and hence favour our results. The highly oxidized V nanoparticles, as evidenced by the dominant oxide peaks at 36.06° corresponding to V_2_O_3_ (111) and at 51.06° corresponding to V_2_O_5_ (200) in Fig. 3(a), are not studied further anyway. Rather, this pure V NP XRD pattern confirms that other samples are not oxidized to form V oxides, and hence are in pure metallic form.

**Figure 4:**
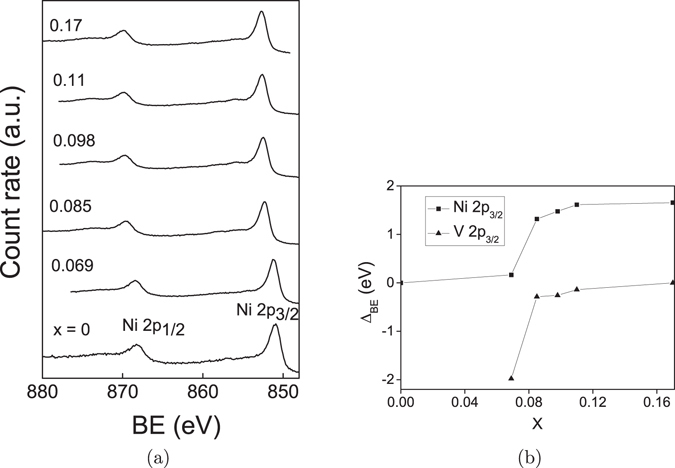
(**a**) High-resolution XPS spectra in Ni 2p region. (**b**) Variation of Ni and V peak positions with *x*.

**Figure 5:**
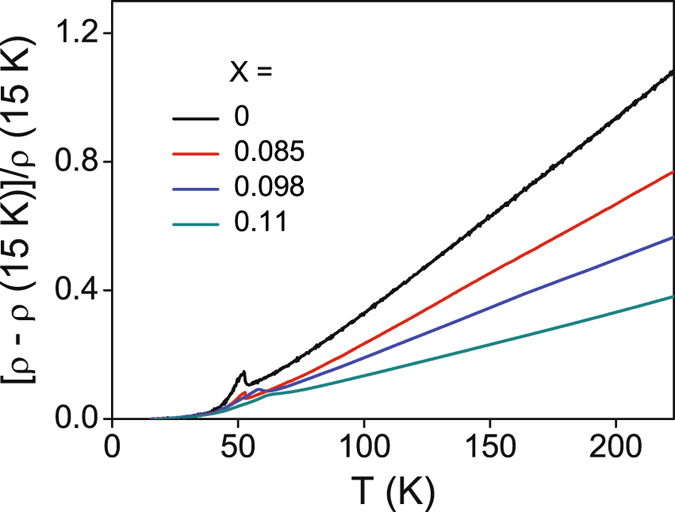
Temperature dependence of the quantity [*ρ* (T) − *ρ* (15 K)]/*ρ* (15 K) in the temperature range 15 K–230 K for *x* = 0.000, 0.085, 0.098 and 0.11.

A further check on the formation of Ni-V alloy solid solution and on whether there are oxide impurities in the samples has been done by X-ray photoelectron spectroscopy (XPS) measurements. The survey XPS spectra of all the samples are shown in Fig. S3(a) of the Supplementary information. The presence only of Ni and V peaks in the spectra, apart from the adventitious C 1*s* peak at 284.5 eV and one O (1*s*) peak at 531 eV, shown later not to participate in any oxide formation except in the pure V nanoparticle case, corroborates the XRD results on the high purity of the pure Ni and alloy nanoparticles. High-resolution XPS spectra in Ni and V binding energy (BE) regions are also taken and displayed in Figs 4(a) and S3(b), respectively. As can be seen, the non-deconvolutable single spin-orbit split peaks in each case confirm the absence of any oxide in the samples. Further, the peaks shift towards higher BE with respect to the pure Ni NP values on increasing *x* in both the sets. The peak shifts (Δ_B*E*_) are plotted as a function of *x* in Fig. 4(b). Both the Ni and V peaks can be seen to vary monotonically and essentially concurrently with *x*, confirming the Ni-V alloy formation with different V concentrations, as also reported earlier^13, 29^. Further, to ensure that the samples are really metallic as required, electrical resistivity *ρ* has been measured as a function of temperature on tightly pressed pellets of some representative NP’s. As the coating diethanolamine is a soft organic compound, it is supposed to get fractured on the application of pressure, making global electrical contact throughout the the NP assembly. The quantity [*ρ* (T) − *ρ* (15 K)]/*ρ* (15 K), which gives a quick comparison for all the samples as all the curves start from zero, has been plotted as a function of temperature for four representative samples in Fig. 5. The values of *ρ* (15 K), which can be taken as a measure of the residual resistivity, for *x* = 0, 0.085, 0.098 and 0.11 are 0.22, 0.28, 0.46 and 1.18 m Ω - cm, respectively. The *x*-variation of this quantity would have contributions from structural defects inherent to NP’s and from increasing amount of V impurity, which can not be decoupled. However, this is not of importance because the main objective of having metallicity is evident from the high-*T* linear increase of resistivity of each sample. The peaks at ~50 K in all the curves may be due to the likely presence of the insulating surfactant in the inter-particle interstitials. However, this would not affect investigations on QGP which have to be explored at much lower temperatures. The above results thus establish that the samples are of desirably satisfactory quality for further investigations pertaining to the exploration of QGP.

**Figure 6:**
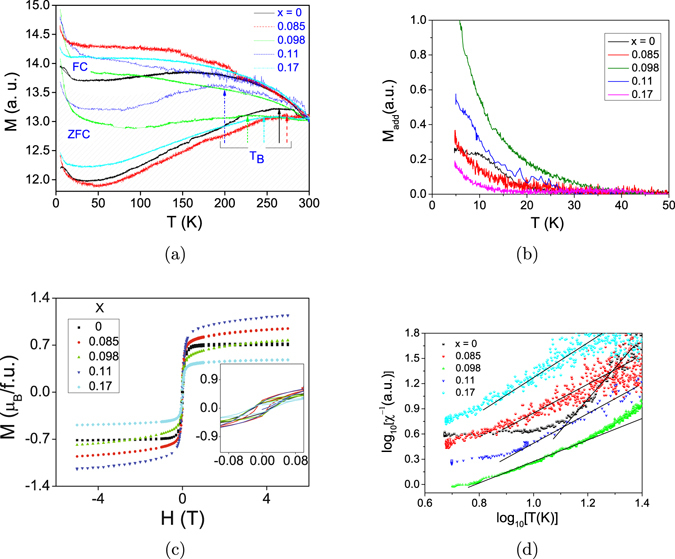
(**a**) FC and ZFC magnetizations versus temperature at 500 Oe field for *x* = 0.000, 0.085, 0.098, 0.11 and 0.17. The curves have been vertically shifted for clarity. The region containing essentially the ZFC curves is hatched for visual separation between FC and ZFC curves. Approximate blocking temperature (*T*_*B*_) is also marked for each case. (**b**) *M*_*add*_ versus *T* curve. (**c**) M-H curves at 2 K for *x* = 0.000, 0.085, 0.098, 0.17 and at 5 K for *x* = 0.11. Inset: Low-field region of the M-H curves showing hystereses. (**d**) $\mathrm{Log}({\chi }_{add}^{-1})$ versus log(*T*) plots in 5 K–25 K region. Since the data in this plot are derived from (**a**) with vertical shifts, they are also shifted accordingly in this figure.

In order to explore the existence of a QGP in the system, we next perform temperature and field *H* dependent DC magnetization *M* and temperature dependent heat capacity measurements as described in ‘Methods’ section later. ZFC and FC *M* − *T* curves in the range 5 K ≤ *T* ≤ 300 K are plotted in Fig. 6(a) for all the samples. The curves are suitably shifted to coincide roughly at the same point at 300 K. The occurrence of ZFC-FC splitting in all the cases is characteristic of magnetic nanoparticles as discussed above^14, 15^, and indicates that all the nanoparticles (with V concentration as high as 17%) have a major magnetic component. This is in agreement with the HRTEM observation of particle agglomeration as pointed out earlier. There is a broad peak in the ZFC curve for each sample and the ZFC-FC splitting takes place at a temperature higher than the (broad) peak value. This is indicative of a distribution of particle size in every sample, as has been discussed earlier^14, 15^, and is also evident from the FESEM analyses (Fig. S1). Figure 6(a) marks the tentative average positions of *T*_*B*_ (ranging between 200 to 250 K) for each sample. Using the relation *T*_*B*_ ≈ *KV*/(30 *k*_*B*_) mentioned above and taking *K* = 5 × 10^4^ erg cm^−1^ for Ni^24^, the average of the particle sizes for different samples would range between 17 to 20 nm, which is more or less consistent with the FESEM results and is suggestive of single-domain nature of the NP’s in all the cases. The near saturation of all the FC curves below *T*_*B*_, until the curves rises again at even lower temperatures, suggests that the NP ensembles are in a BFM state below *T*_*B*_ and in an SPM state above it, as discussed in the introduction.

**Figure 7:**
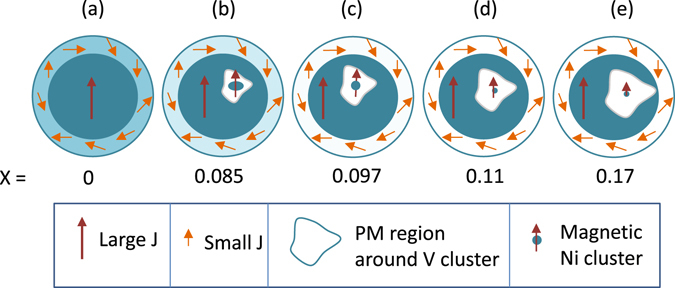
Schematic pictures of nanoparticle magnetic structures at *x* = 0 (**a**), 0.085 (**b**), 0.098 (**c**), 0.11 (**d**) and 0.17 (**e**). Large *J*: spins with large FM exchange interaction (dark cyan) giving rise to a single large moment, small *J*: spins with weak FM exchange interaction (lighter cyan). Lighter shade implies weaker exchange interaction.

Another feature of the *M* − *T* curves which is apparent is the low-*T* upturn below ~20 − 30 K in both FC and ZFC magnetizations of all the samples. This rise would primarily mean the presence of an extra FM or PM-like phase in the NP. In order to determine what exactly the magnetic nature of this phase for a particular *x* is, we first extrapolate the plateau region of the corresponding FC curve down to the lowest temperature measured, and then subtract the extrapolation from the measured FC curve. Figure S4 in the Supplementary information shows the extrapolations, while Fig. 6(b) shows the subtracted curves representing the additional magnetization component *M*_*add*_, for all *x* values. The saturation of *M*_*add*_ for the pure Ni NP sample, as can be seen in Fig. 6(b), suggests that the NP’s in this particular sample have a fractional volume which is still FM, albeit with a lower critical temperature (~20 K). The occurrence of this additional magnetization is in accordance with the features of a generic magnetic NP ensemble, and must be due to the disorder in the NP surface spins with exchange interaction *J*_*s*_ less than the value *J* in the core, as discussed in the introduction. The *M* − *H* curve at 2 K for this sample is shown in Fig. 6(c). The small hysteresis loop in the curve and its saturation at higher fields confirm that both the magnetic components of these NP’s are FM at 2 K. A possible model of the magnetic structure of the pure Ni NP is shown schematically in Fig. 7(a): it consists of an inner FM core with a large exchange interaction and an outer FM shell with smaller exchange interaction. The *M*_*add*_ − *T* curves for all other samples (*x* ≠ 0) diverge down to the lowest measured temperature. If we assume that the curves diverge down to 0 K, it would suggest that a part of the NP volume in these cases is essentially PM in nature. To investigate this PM-like phase further, the log of the inverse susceptibility of the additional phase $\mathrm{log}({\chi }_{add}^{-1})=\,\mathrm{log}(H\mathrm{.}{M}_{add}^{-1})$ is plotted as a function of log(*T*) for all samples in Fig. 6(d). The exponent *λ* occurring in the power-law *χ* ~ *T* ^*λ*−1^ as discussed earlier is then roughly estimated. Let us denote the exponent determined from the magnetization data above 20 K as *λ*_*M*_. The slopes of initial inverse susceptibilities obtained this way and as shown in Fig. 6(d) must then be equal to (1 − *λ*_*M*_). It can be seen from the figure that the susceptibility for *x* > 0 keeps diverging with an exponent 1 − *λ*_*M*_ smaller than that for *x* = 0 in a range of 0.5–1, considering the large possible errors in its determination due to possible errors in background subtraction. The change of the exponent with *x* means that the behaviour of *x* > 0 NP’s is non-universal, and hints at the presence of critical fluctuations associated with QGP in these NP’s^2, 9^. This two-phase (FM + PM-like) coexistence in the *x* ≠ 0 NP’s is corroborated by the small hysteresis loop in the corresponding *M* − *H* curve, along with the non-saturation of the curve at high fields at 2 K (Fig. 6(c)). However, this inference on the presence of QGP in *x* > 0 NP’s is somewhat too qualitative, and hence further investigations to confirm this are desirable. For this, we have measured heat capacity *C*, which is proportional to *c*, as a function of temperature, as described in the ‘Methods’ section later.

**Figure 8:**
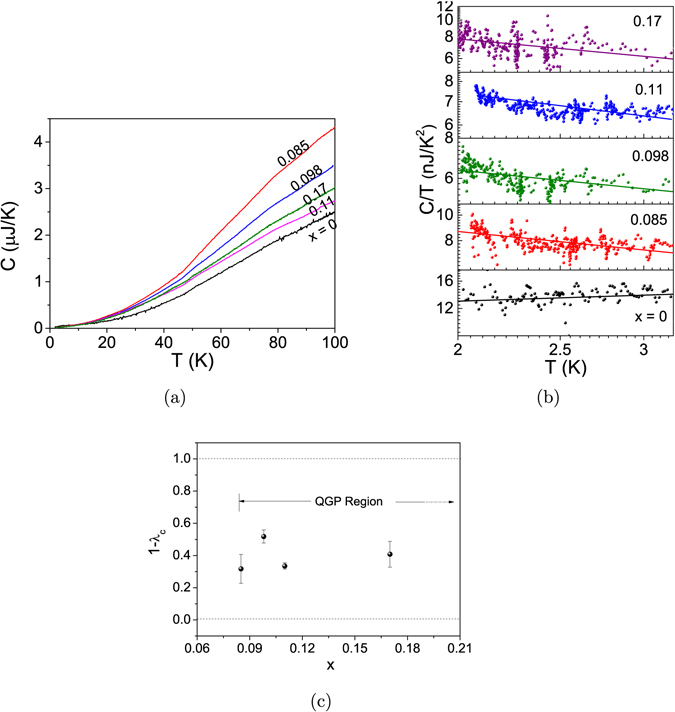
(**a**) *C* − *T* plots in the temperature range 2 K–230 K for *x* = 0.000, 0.085, 0.098 and 0.11. (**b**) Log-log plots of *C*/*T* versus *T* (symbols) in low-T (2 K–3.5 K) range for various *x* values, and their linear fits (solid lines). (**c**) Plot of (1 − *λ*_*C*_) versus *x*. The prospective QGP region is indicated.

Figure 8(a) displays the *C* − *T* plots in the temperature range 2 to 100 K for all the samples. The shapes of the curves are typical of Ni and its alloys, as reported by Alizade and Kerimov^30^. The shapes include the magnetic contributions apart from the contributions from phonons and electrons. The presence of QGP can next be explored by examining the *C* − *T* data at temperatures below ~3 K, in line with the analysis by Westerkamp *et al*.^31^. It is achieved by plotting ${\rm{log}}(\frac{C}{T})$ as a function of log(*T*), as shown in Fig. 8(b). A literature survey suggests that specific heat of solids has a phonon component *c* ~ *T* ^3^ dominant at rather high temperatures^32^, a low-temperature linear (*c* ~ *T*) electronic component in case of metals^32^ and in some magnetic insulators^33^, and a *c* ~ *T* ^−2^ term below 1 K if there are hyperfine interactions^34, 35^. So, in the (low) temperature range 2 K < *T* < 3 K, $\frac{C}{T}$ should be constant for a normal metal exhibiting universal behaviour. Considering the relation *c* ~ *T* ^*λ*^, i.e., $\frac{C}{T}$ ~ *T* ^*λ*−1^, and denoting *λ* in the case of *C* − *T* measurements by *λ*_*C*_, this will lead to the slope (1 − *λ*_*C*_) of the $\mathrm{log}(\frac{C}{T})$ versus log(*T*) curve equal to zero. For the pure Ni NP’s (*x* = 0) where both the components are FM as described above, 1 − *λ*_*C*_ ~ 0 is therefore expected. This is verifiable from the very slightly positive slope (0.04) of the corresponding curve in Fig. 8(b); the slight positive slope must be due to the phonon contribution. The curves for *x* > 0 NP’s, however, have clear low-*T* upturns indicative of fractional values of *λ*_*C*_. Such a low-*T* upturn in a $\mathrm{log}(\frac{C}{T})$ versus log(*T*) curve can have origins that can be classified into two categories: (i) magnetic defects^36^, and (ii) material-specific properties, like QGP^9^, low-*T* state of a specific insulator o-TaS_3_^37^ and magnetically frustrated systems^38^. In the present Ni_1−*x*_V_*x*_ system, however, presence of critical fluctuations associated with a QGP seems to be most relevant. This supplements the inference from the *M* − *T* measurements, as discussed earlier, that these NP’s possess a QGP.

In the scenario of the nanoalloy system possessing a QGP, it is imperative to examine also the *x*-dependence of *λ*_*C*_, or of (1 − *λ*_*C*_) for that matter, for *x* > 0 NP’s which exhibit fractional values of *λ*_*C*_. For this, the $\mathrm{log}(\frac{C}{T})$ versus log(*T*) data has to be fitted in the negative slope region for *x* > 0. Assuming the background coming from the universal electronic contribution to be the same, the slopes taken directly from the curves would approximately represent the correct *λ*_*C*_ values. Further, it is to be noted that the relevant data are quite scattered due to the extremely low intensity in this region, and are in a narrow temperature range. Therefore, claiming a good fit to the data would not be quite correct. However, a tentative fit, albeit with large error bars, is feasible. The plot of (1 − *λ*_*C*_) versus *x* determined in this manner for *x* > 0 is shown in Fig. 8(c). This curve can be compared with the results by Ubaid-Kassis *et al*.^9^, wherein the QGP region is identified as the composition range having (1 − *λ*) values between 0 and 1. In the present case, the QGP region can be considered to range from *x* = 0.085, where the upturns hinting at the emergence of critical fluctuations start, to an *x*-value far above *x* = 0.17, where one would expect a complete breakdown of ferromagnetism in the NP’s due to the presence of a large amount of V atoms.

These results can be understood from the following model of the Ni_1−*x*_V_*x*_ NP’s. The *x* = 0 case has been discussed already: such a NP can be considered to consist of an FM core with exchange interaction *J*, surrounded by a surface FM layer with exchange interaction *J*_*s*_ < *J*. On introducing V in Ni, PM clusters surrounding the V atoms form an infinite chain (infinite PM cluster) in an NP, but at a much smaller percolation threshold, i.e. the V concentration, than in the bulk. It should be noted that percolation thresholds are known to be reduced drastically on size reduction of the particle constituting the matrix^39^. This way, a significant volume of the NP would be left with all-Ni atoms after the infinite PM cluster, having a possibility to include even smaller FM Ni zones, is formed. A schematic of the percolation structure of *x* = 0.085 NP, which is just beyond this reduced threshold, is shown in Fig. 7(b). The large PM zones here are represented by a white enclosed region in the core. The arms of the percolation network including this cluster would certainly extend up to the NP surface, but are not shown. As argued in the introduction, there is a statistical probability of occurrence of smaller FM cluster(s), represented by a small coloured circle with arrows, inside this large PM cluster. These FM Ni-clusters within the PM cluster fluctuate and are responsible for the low-*T* upturn in the susceptibility and hence for the presence of QGP^9^ in the *x* > 0 NP’s. The arms of the PM cluster extending the surface would reduce the Ni moments there, effectively making the NP surface less FM and more PM. The remaining Ni-rich region in the NP is still FM and is of sub-domain dimensions, thereby retaining still the (complex) magnetic behaviour of a NP. This is why the magnetization still consists of the NP-like ZFC and FC features. On further addition of V, the NP surface would become more PM, the paramagnetic zones would grow in size, and the FM Ni inclusions would shrink, as shown in Fig. 7(c–e). Thus, the present findings in the scenario of the NP system possessing a QGP suggest that the QGP phase in materials in nanodimensions constitutes a partial volume of the NP, the rest still remaining normal magnetic NP phase. This is in contrast to bulk Ni_1−*x*_V_*x*_^9^, wherein the whole sample volume in the QGP regime is essentially PM with FM cluster inclusions, and can be considered as a single, uniformly distributed QGP.

## Conclusion

Ni_1−*x*_V_*x*_ (0 ≤ x ≤ 0.17) nanoalloys of mean diameters 18–33 nm are prepared by a chemical reflux method. The compositions of the finally synthesized nanoalloys are determined using EDAX. The particle sizes are calculated from FESEM images, while HRTEM images and SAED patterns display magnetic and crystalline structure of the nanoparticles, respectively. The magnetic nature is later confirmed by *M* − *T* and *M* − *H* measurements. Further, XRD and XPS spectra confirm that the nanoalloys are indeed a solid solution of Ni and V, without any trace of oxides. The temperature dependence of resistivity confirms the metallic nature of the NP’s and finally establishes that the samples are of satisfactory quality for QGP studies. The *M* − *T* curves of all the samples exhibit magnetic nature characteristic of magnetic nanoparticles with a size distribution. However, each *M* − *T* curve possesses an additional feature - the low temperature upturn. For *x* > 0, the additional susceptibility characterizing this upturn has a fractional power-law temperature dependence. The finding is further confirmed by a fractional power-law temperature dependences of *C*/*T* for *x* > 0 NP’s as well. These two synergistic observations are suggestive of the presence of a QGP for all *x* > 0 NP’s studied. The QGP region has been identified to range from *x* = 0.085 to *x* ≫ 0.17 where complete destruction of ferromagnetism of NP’s is expected. A percolation-based model is proposed to explain the results in line with Ubaid-Kassis *et al*.’*s* ^9^ report. Finally, the anticipated QGP in the nanoparticle system is different from that in the bulk as the former is accompanied with a leftover fractional volume having normal magnetic NP properties.

## Methods

The nanoparticles of Ni_1−*x*_V_*x*_ (*x* = 0, 0.069, 0.085, 0.098, 0.11, 0.17 and 1) were synthesized by reduction of metal precursor salts vanadium (III) chloride (VCl_3_.H_2_O) and nickel (II) chloride (NiCl_2_), either separately for the elemental cases or simultaneously with appropriate stoichiometry for the nanoalloys, by hydrazine hydrate in the presence of the surfactant diethanolamine in a conventional reflux apparatus^13^. In the cases of elemental nanoparticles, typically 0.5 mmol of VCl_3_ (NiCl_2_) was dissolved in 30 ml distilled water to yield complexes of V^2 +^ (Ni^2+^) ions in the solution; for nanoalloys, proportionately appropriate amounts of the two salts were dissolved sequentially in distilled water. Subsequently, 5 ml of diethanolamine was added as a surfactant to the above solution, followed by 6 ml of hydrazine hydrate as the common reducing agent. Finally, 40 ml distilled water was added to this, and the resulting solution was refluxed for 8 h at 110° C in an oil bath. The black-colored precipitate, i.e., the alloy, was then washed with warm distilled water, centrifuged at 3500 RPM and dried in vacuum for 48 h.

The morphologies of the nanoalloys were investigated by ZEISS SUPRA 40 field-emission scanning electron microscope and JEOL JEM-2100 high resolution transmission electron microscope operated at 200 kV. A drop of the colloidal nanoparticles, pre-sonicated in acetone, was placed on a small quartz peace to prepare the sample for field-emission scanning electron microscopy (FESEM); the drops were placed on a carbon supported Cu transmission electron microscope grid for high resolution transmission electron microscopy (HRTEM) and selected area electron diffraction (SAED). Energy dispersive X-ray analyses (EDAX) of the nanoalloys were performed using a JEOL scanning electron microscope to determine the final synthesized composition *x*_*s*_. The phases were determined by X-ray diffraction (XRD) on a Philips X’Pert MRD system using Cu K _*α*_ radiation operated at 45 kV and 40 mA. The stoichiometries of the samples were further studied using X-ray photoelectron spectroscopy (XPS). XPS spectra were recorded on a PHI 5000 Versaprobe II system using a micro-focused monochromatic Al *K*_*α*_ source (*hν* = 1486.6 eV), a hemispherical analyser, and a multichannel detector. Charge neutralization in each measurement was achieved using a combination of low energy Ar^+^ ions and electrons. The binding energy scale was charge referenced to C 1 s peak at 284.5 eV. High-resolution XPS spectra were acquired at 58.7 eV analyzer pass energy in steps of 0.25 eV. For the physical property measurements, only some representative samples out of all the prepared ones were chosen. The temperature dependences of sample resistivities in the temperature range 15–300 K and at zero field were measured on pelletized nanoalloys by four-probe technique using a Lakeshore Resistivity 7500 set-up with the help of a nanovoltmeter as a current source. DC magnetizations versus temperature (5 K ≤ T ≤ 300 K) at 500 Oe and versus field (−5 T ≤ H ≤ 5 T) at 2 K were measured using a cryogen-free 9 T CRYOGENIC physical property measurement system (PPMS) and a Quantum Design MPMS SQUID VSM EverCool system. Heat capacity measurements were finally performed using the same PPMS system.

## Electronic supplementary material

Exploring quantum Griffiths phase in Ni1−xVx nanoalloys: supplementary information
