# Supplementary material for: Exploring quantum Griffiths phase in Ni1−xVx nanoalloys
Source: Sci Rep. 2017 Apr 21;7:1223. doi: 10.1038/s41598-017-01423-x (PMC5430624; doi:10.1038/s41598-017-01423-x)
Supplement: Supplementary file 1 — Exploring quantum Griffiths phase in Ni1−xVx nanoalloys: supplementary information [file 41598_2017_1423_MOESM1_ESM.pdf]

Exploring quantum Griffiths phase in $Ni_{1-x}V_x$ nanoalloys: supplementary information

PRIYADARSINI SWAIN[1], **SUNEEL K. SRIVASTAVA**[2], **AND SANJEEV K. SRIVASTAVA**[1,*]

[1] *Department of Physics, Indian Institute of Technology Kharagpur, Kharagpur - 721302, India*
[2] *Department of Chemsitry, Indian Institute of Technology Kharagpur, Kharagpur - 721302, India*
[*] *sanjeev@phy.iitkgp.ernet.in*

Compiled February 6, 2017

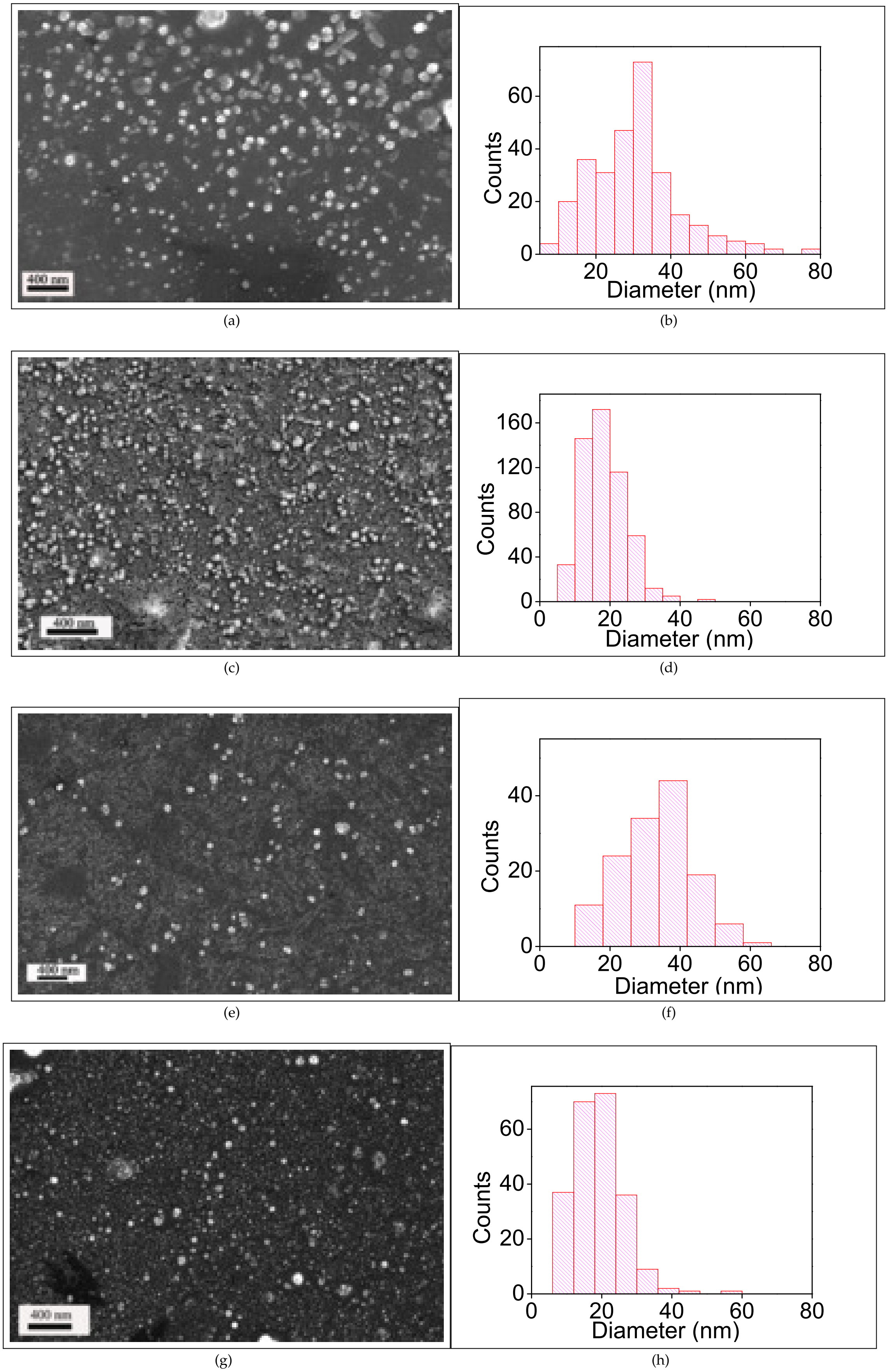

Fig. S1. FESEM images of $Ni_{1-x}V_x$ samples with V compositions x = 0.000 (a), 0.085 (c), 0.098 (e) and 0.11 (g). The corresponding particle size distributions are shown in (b), (d), (f) and (h), respectively.

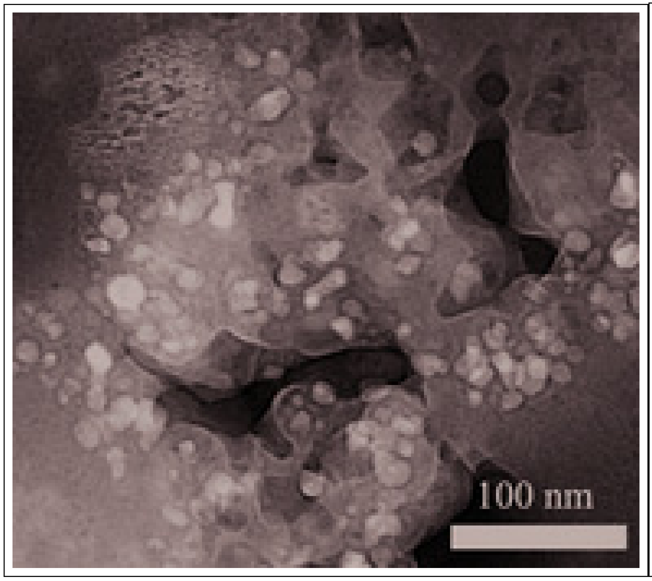

(a)

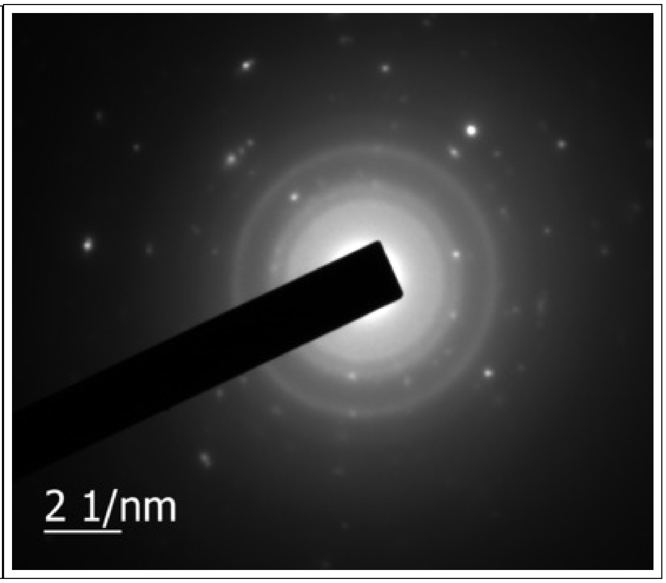

(b)

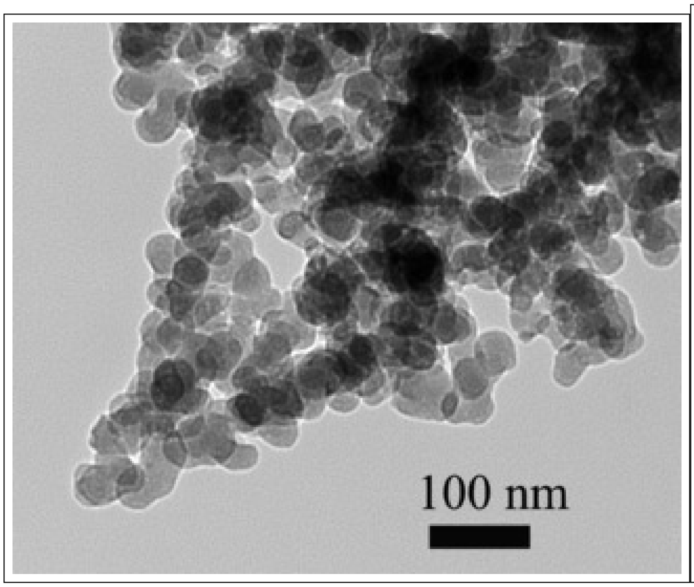

(c)

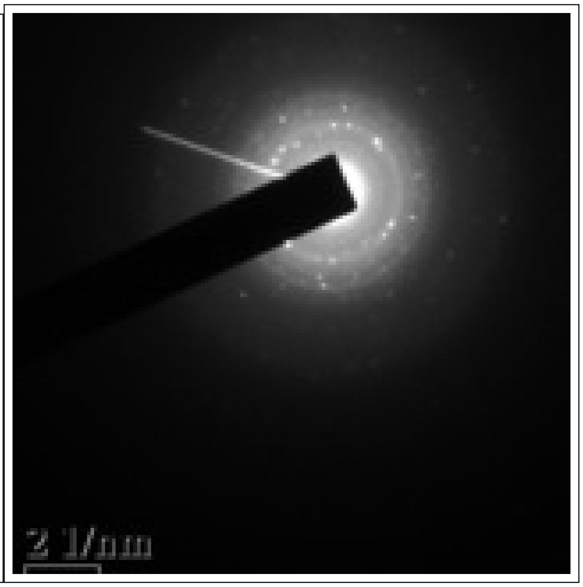

(d)

Fig. S2. HRTEM images of $Ni_{1-x}V_x$ samples with V compositions x = 0.098 (a) and 0.11 (c). The corresponding SAED patterns are shown in (b) and (d), respectively.

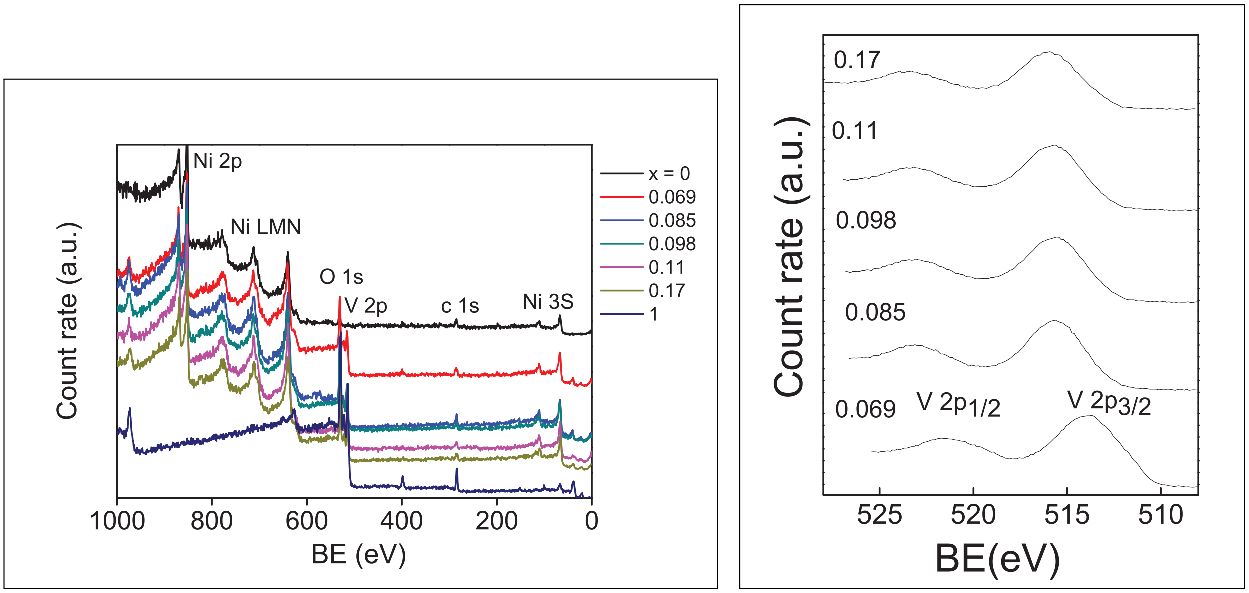

(a) (b)

Fig. S3. (a) Survey XPS spectra of $Ni_{1-x}V_x$ samples. (b) High-resolution XPS spectra in V 2p region.

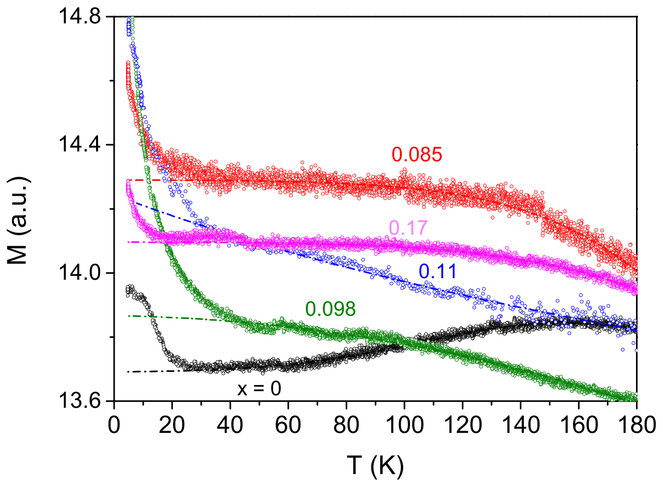

Fig. S4. FC magnetization data in 5 K - 180 K temperature range. Dashed lines are the extrapolated curves which have been subtracted from the corresponding FC magnetizations to get the magnetization M_{add} of the additional component of the NP's.
